# Artificial Intelligence CT Texture Radiomics for Outcome Prediction After EVAR: A Narrative Review

**DOI:** 10.3390/diagnostics16070964

**Published:** 2026-03-24

**Authors:** Chiara Zanon, Giovanni Alfonso Chiariello, Tommaso D’Angelo, Emilio Quaia

**Affiliations:** 1 Department of Radiology, University of Padova, Via Giustiniani 2, 35128 Padova, Italy; 2 Department of Cardiovascular Sciences, Agostino Gemelli Foundation Polyclinic IRCCS, 00136 Rome, Italy; 3 Diagnostic and Interventional Radiology Unit, BIOMORF Department, University Hospital “Policlinico G. Martino”, 98125 Messina, Italy

**Keywords:** radiomics, texture analysis, endo vascular aneurysm repair, abdominal aortic aneurysm, artificial intelligence, computed tomography

## Abstract

**Background**: Endovascular aneurysm repair (EVAR) requires lifelong imaging surveillance because endoleaks, aneurysm sac expansion, and severe adverse events occur in up to one-third of the patients. Conventional follow-up based on sac diameter and visual assessment may fail to detect early microstructural changes that precede clinical deterioration. **Methods**: This narrative review summarizes the current evidence on texture-based radiomics and artificial intelligence (AI) applied to computed tomography (CT) and CT angiography (CTA) for post-EVAR outcome prediction and surveillance. Original studies evaluating radiomic features and AI-based models for endoleak detection, aneurysm sac behavior, and EVAR-related adverse events were included and qualitatively synthesized. **Results**: Ten studies were included. Radiomic features describing texture heterogeneity, gray-level nonuniformity, entropy, and spatial complexity were extracted from the aneurysm sac, intraluminal thrombus, and perivascular adipose tissue. Machine learning and deep learning models achieved good to excellent performance, with reported AUC values ranging from 0.78 to 0.95 for predicting endoleaks, sac expansion, and severe adverse events. Texture-based radiomics consistently outperformed morphology-only assessments and showed complementary value to deep learning, including applications on non-contrast CT. **Conclusions**: CT texture radiomics combined with AI represents an emerging research approach with potential relevance for post-EVAR surveillance, although current evidence remains limited. By capturing tissue heterogeneity beyond conventional morphology, radiomics may enable the earlier detection of complications and support risk-adapted follow-up. However, the heterogeneity of methods limited external validation, and reproducibility issues remain major barriers to clinical translation.

## 1. Introduction

**Figure 1:**
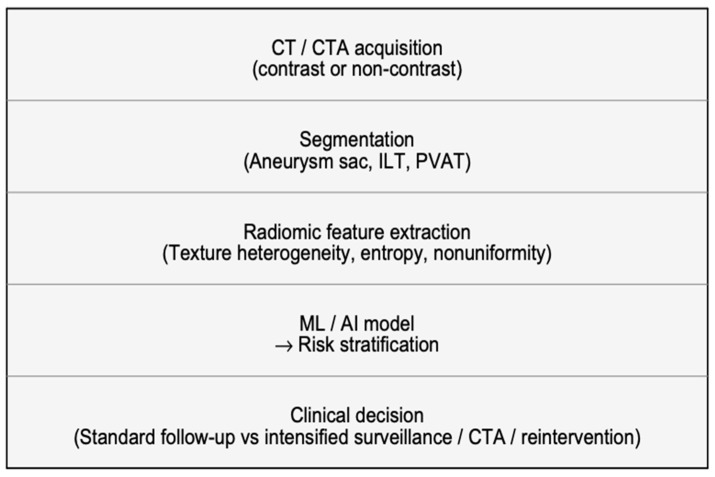
Proposed radiomics-driven workflow for post-EVAR surveillance. CT/CTA acquisition (contrast-enhanced or unenhanced) is followed by segmentation of predefined regions of interest (e.g., aneurysm sac, lumen/intraluminal thrombus, and perivascular adipose tissue), radiomic feature extraction, machine learning/deep learning model development (the arrow indicates that the model output is used for risk stratification), and output-based risk stratification to support clinical decision-making (standard vs. intensified surveillance and/or reintervention).

Endovascular aneurysm repair (EVAR) is currently the most frequently adopted treatment for abdominal aortic aneurysm (AAA), offering reduced perioperative morbidity and faster recovery compared with open surgery [1]. Despite these advantages, long-term outcomes remain variable: endoleaks, persistent endotension, aneurysm sac non-regression or enlargement, and EVAR-related severe adverse events are reported in a substantial proportion of patients, with rates commonly approaching 30–35% during follow-up [2]. Consequently, lifelong imaging surveillance is recommended to detect complications and guide reinterventions [3]. Computed tomography angiography (CTA) is the reference imaging modality for post-EVAR assessment, as it provides a high spatial resolution and reliable evaluation of stent graft integrity, endoleaks, and sac diameter changes [4]. However, standard surveillance strategies rely predominantly on morphological endpoints, maximum sac diameter, and volume, together with qualitative visual interpretation [4]. These conventional metrics may lag behind the underlying biological processes that drive post-EVAR evolution and may not fully capture subtle microstructural alterations preceding clinically evident sac growth [5]. Moreover, repeated contrast-enhanced CTA may be problematic in frail patients due to radiation burden and the risk of contrast-induced nephrotoxicity, motivating interest in advanced, noninvasive imaging biomarkers, including approaches applicable to non-contrast CT [6]. Radiomics has emerged as a quantitative imaging paradigm enabling the extraction of high-dimensional features from routinely acquired CT images [7]. In particular, texture-based radiomics characterizes gray-level distribution and spatial relationships within regions of interest, providing objective descriptors of heterogeneity, entropy, nonuniformity, and complexity that extend beyond what is appreciable by visual assessment [8]. In vascular imaging, these texture signatures may act as surrogates of clinically relevant processes such as thrombus remodeling, subtle endoleak-related flow effects, wall degeneration, and periaortic inflammatory activity, potentially anticipating morphological changes [9]. When combined with machine learning (ML) or deep learning (DL), radiomic features can support predictive models for sac behavior (regression versus stability/expansion), aneurysm growth, endoleak detection, and the risk of EVAR-related severe adverse events [10] (Figure 1). In recent years, multiple studies have explored texture radiomics extracted from different anatomical targets, including the aneurysm sac, intraluminal thrombus, and perivascular adipose tissue, reporting encouraging diagnostic and prognostic performance [11]. Nonetheless, the published evidence remains heterogeneous with respect to imaging protocols, segmentation strategies, feature extraction pipelines, model development, and validation approaches, and several barriers still limit clinical translation, including reproducibility and external validation. Therefore, the aim of this narrative review supported by a structured literature search is to summarize and critically discuss current evidence on texture-based radiomics and AI applied to CT/CTA after EVAR.

From a clinical perspective, the primary use case of CT texture radiomics in the post-EVAR setting is risk-stratified surveillance. Specifically, radiomics and AI models are being investigated for three main applications: (i) the automated or assisted detection of endoleaks, including on non-contrast CT; (ii) the early prediction of aneurysm sac behavior, particularly sac expansion or lack of regression; and (iii) the prediction of EVAR-related adverse clinical outcomes that may require closer monitoring or reintervention. Rather than guiding primary treatment planning, the current evidence mainly supports the role of radiomics as a decision-support tool for post-procedural surveillance and individualized follow-up strategies.

Unlike broader reviews addressing artificial intelligence applications across multiple vascular diseases and imaging modalities, the present work specifically focuses on CT-based texture radiomics in patients undergoing EVAR. By concentrating on a single clinical scenario and imaging technique, this review aims to provide a more focused synthesis of studies investigating radiomic biomarkers for post-EVAR surveillance, including endoleak detection, the prediction of aneurysm sac behavior, and risk stratification for EVAR-related adverse events.

## 2. Texture Analysis

**Figure 2:**
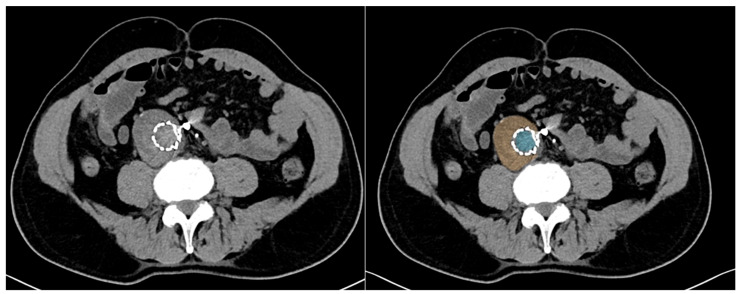
Axial CT images acquired after EVAR without contrast medium. On the left, the same slice is shown after segmentation of the lumen (light blue) and aneurysm sac (orange), defining the regions of interest for radiomic feature extraction and subsequent AI-based analysis.

Texture analysis in computed tomography (CT) is a quantitative imaging technique that characterizes the spatial distribution and relationships of gray-level intensities within an image, extending beyond conventional visual assessment and simple morphological measurements [12]. By applying mathematical models such as the gray-level co-occurrence matrix (GLCM), gray-level run length matrix (GLRLM), and gray-level dependence matrix (GLDM), texture analysis captures image features related to heterogeneity, uniformity, entropy, and spatial complexity [13]. These features reflect the underlying tissue microstructure, including variations in composition, perfusion, inflammation, and fibrosis, which may not be visually appreciable. In vascular imaging, CT texture analysis enables a detailed characterization of the aneurysm sac, intraluminal thrombus, and perivascular tissues [13]. When combined with machine learning or artificial intelligence approaches, texture-derived features can identify subtle patterns associated with disease progression, treatment response, and clinical outcomes [14]. As a result, CT texture analysis represents a powerful noninvasive biomarker, supporting risk stratification and personalized imaging-based decision-making in modern radiology [14] (Figure 2).

## 3. Methods

### 3.1. Search Strategy

This study was designed as a narrative review supported by a structured literature search rather than a formal systematic review or meta-analysis.

A structured literature search was conducted in PubMed/MEDLINE, Scopus, and Web of Science to identify studies investigating radiomics, texture analysis, or artificial intelligence applied to CT or CTA in patients undergoing endovascular aneurysm repair (EVAR). The search included all the eligible articles published up to 7 January 2026. The following combination of keywords was used: (“endovascular aneurysm repair” OR EVAR) AND (“abdominal aortic aneurysm” OR AAA) AND (radiomics OR “texture analysis” OR “artificial intelligence”) AND (“computed tomography” OR CT OR CTA). The reference lists of the included articles were also screened to identify additional relevant studies.

### 3.2. Eligibility Criteria

Studies were included if they were original research articles evaluating radiomics, texture analysis, and/or artificial intelligence models derived from CT or CTA in the post-EVAR setting, reporting a defined clinical endpoint (e.g., endoleak detection, aneurysm sac behavior, aneurysm growth, or EVAR-related adverse events) together with quantitative performance metrics.

**Figure 3:**
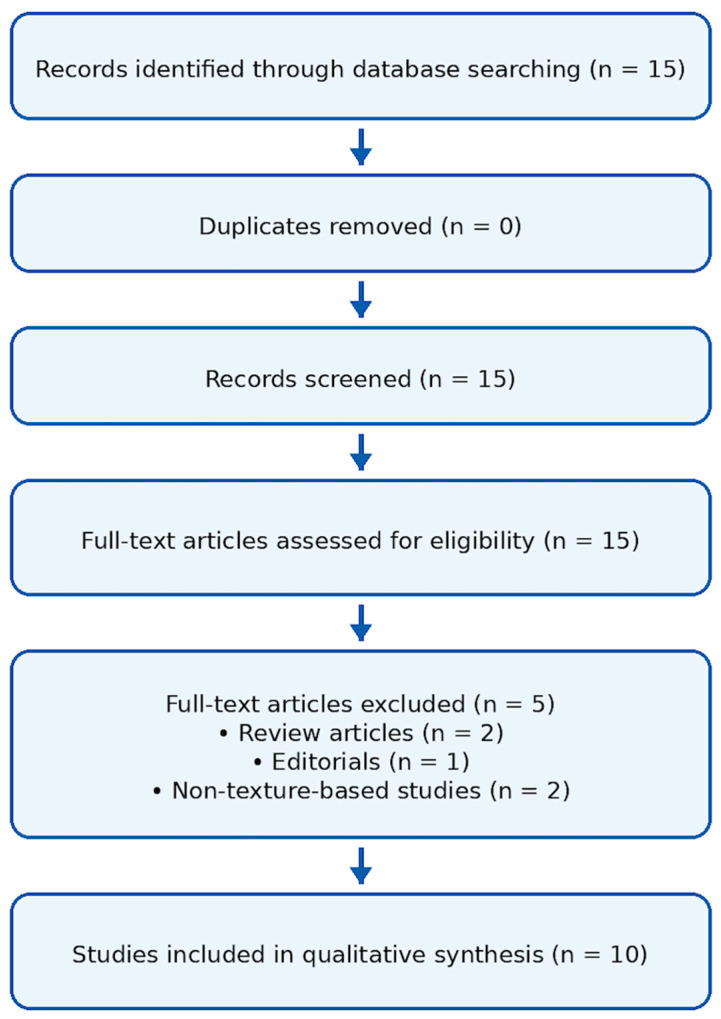
Flow diagram showing study identification (*n* = 15), screening, full-text eligibility assessment, exclusions (*n* = 5: reviews *n* = 2, editorials *n* = 1, non-texture-based studies *n* = 2), and final inclusion in qualitative synthesis (*n* = 10).

Articles were excluded if they were reviews, editorials, conference abstracts, non-CT-based imaging studies, studies not involving radiomics/texture analysis or AI approaches, or studies without outcome-related analyses. A flow diagram of the study selection process is provided in Figure 3.

### 3.3. Data Extraction and Qualitative Synthesis

Two authors independently screened titles/abstracts and full texts. Extracted variables included sample size, imaging modality, radiomic targets, feature extraction and selection strategies, AI approach, validation scheme, and diagnostic/prognostic performance (AUC, sensitivity, specificity, Dice, etc.). Given the heterogeneity across studies, the findings were synthesized qualitatively. Due to the substantial heterogeneity across studies, including differences in CT acquisition parameters, image preprocessing workflows, segmentation approaches, radiomic feature extraction pipelines, and machine learning model development, the results summarized in Table 1 should be interpreted as a descriptive overview rather than a direct quantitative comparison. The table is intended to highlight key methodological characteristics and reported performance metrics across studies, while acknowledging that methodological variability limits strict cross-study comparability. In addition, the cross-study comparison of reported performance metrics should be interpreted with caution. AUC values alone do not fully capture study comparability because the included studies differ substantially in cohort size, endpoint definition, validation design, and overall risk of bias. Accordingly, Table 1 is intended as a structured descriptive summary rather than a basis for the direct ranking of model performance across studies (Figure 3. Table 1).

## 4. Results

### 4.1. Detection and Characterization of Endoleaks

Texture-based radiomics and machine learning have emerged as powerful methods for endoleak detection after EVAR. By quantifying CT attenuation and heterogeneity, these methods improve diagnostic accuracy on both contrast and non-contrast CT and enable the identification of clinically aggressive endoleaks, supporting more effective post-EVAR surveillance and risk stratification.

Yang et al. developed a texture-informed deep learning framework for detecting endoleaks after EVAR using non-contrast CT. The retrospective cohort included 167 patients (85 with endoleak, 82 without). After aneurysm sac segmentation, radiomic texture features describing CT attenuation, density dispersion, and heterogeneity were extracted and compared between groups. Endoleak sacs demonstrated higher mean CT values and a greater texture dispersion than non-endoleak sacs. These texture differences informed the training of a deep learning segmentation model that directly predicted endoleak regions. In the validation set, the model achieved an AUC of 0.951, Dice similarity coefficient of 0.814, sensitivity of 0.877, and specificity of 0.884. The results indicate that texture and density patterns on non-contrast CT can reliably support automated, high-performance endoleak detection after EVAR [17].

Hu et al. evaluated texture-based radiomics from non-contrast CT for detecting endoleaks after EVAR. The retrospective study included 216 AAA patients (mean age 69  ±  8 years, 191 men), of whom 64 (29.6%) had endoleaks. From non-contrast CT, 1955 radiomic features describing intensity, texture heterogeneity, and dispersion were extracted from the aneurysm sac. Patients with an endoleak showed a higher attenuation (41.7 vs. 33.6 HU, *p* < 0.001) and lower texture dispersion (51.5 vs. 58.8, *p* < 0.001) than the controls. Twelve ML models achieved a mean AUC of 0.86  ±  0.05, accuracy of 81%  ±  4, sensitivity of 88%  ±  10, and specificity of 78%  ±  5. At >90% sensitivity, the specificity remained 72%  ±  10, supporting texture-based non-contrast CT for endoleak detection [18].

Charalambous et al. investigated whether texture-based radiomic features (RFs) from CTA can identify aggressive type 2 endoleaks (T2EL) after EVAR. In a prospective cohort (2018–2020), patients with T2EL on 1-month CTA were followed at 6 months and 1 year and classified by aneurysm sac growth. From 944 initial texture-rich RFs (including heterogeneity, gray-level variation, and spatial complexity), 58 features at 1 month and 51 at 6 months were selected using AI-driven feature reduction. Support vector machine models achieved a strong predictive performance: 1-month RFs predicted sac expansion with an AUC of 89.3%, sensitivity of 100%, and specificity of 78.6%, while 6-month RFs reached an AUC of 95.5%, sensitivity of 100%, and specificity of 90.9%. The results highlight CTA texture patterns as biomarkers of aggressive T2EL behavior [10].

García et al. investigated the value of CT texture analysis for characterizing post-EVAR aneurysm evolution related to endoleaks. Texture features were extracted from regions of interest within the aneurysmatic intraluminal thrombus on CTA images of patients with different post-procedural outcomes. Three texture methods were applied, the gray-level co-occurrence matrix (GLCM), gray-level run length matrix (GLRLM), and gray-level difference method (GLDM), to quantify heterogeneity and spatial gray-level patterns. Texture analysis demonstrated a strong discriminatory ability between favorable and unfavorable post-EVAR evolution, outperforming visual CTA assessment alone. Among the methods, GLCM achieved the highest classification accuracy (93.4%), followed by GLRLM (90.2%) and GLDM (82.0%). These findings support CT texture features as complementary biomarkers for endoleak-related outcome assessment after EVAR [20].

### 4.2. Prediction of Aneurysm Growth and Disease Progression

Texture-based radiomics has shown promise for predicting aneurysm growth and disease progression after EVAR. By analyzing texture heterogeneity from the aneurysm sac and perivascular adipose tissue on CT, these studies demonstrate that early radiomic features outperform conventional clinical and morphological parameters in identifying aneurysms at risk of post-EVAR expansion and progressive instability.

Lv et al. investigated the texture-based radiomic features of perivascular adipose tissue (PVAT) as predictors of abdominal aortic aneurysm (AAA) growth after EVAR. The retrospective cohort included 79 patients (mean age 68 ± 9 years, 89% men), with 19 patients (24%) showing aneurysm growth on follow-up CT. From PVAT, 107 radiomic features describing texture heterogeneity, dependence, dispersion, and shape complexity were extracted. Growing AAAs demonstrated a higher surface area-to-volume ratio (0.70 vs. 0.63, *p* = 0.04) and more heterogeneous texture patterns with a lower dependence and higher dispersion (*p* < 0.05). The integrated clinico-radiological model achieved an AUC of 0.78 (95% CI 0.65–0.91) and specificity of 87%, outperforming radiomics-only and clinical-only models (AUC 0.69 each). These findings highlight PVAT texture as a biomarker of post-EVAR aneurysm progression [16].

Ding et al. evaluated whether early postoperative CT texture features can predict later aneurysm progression after EVAR. The study included 99 infra-renal AAA patients who underwent serial CT scans between 2014 and 2019. A texture analysis of the aneurysm sac was performed using GLCM, GLRLM, and GLDM, focusing on gray-level relationships, run-length patterns, and intensity differences. A multilayer perceptron neural network was used for classification. No significant differences were observed in clinical or morphological variables between the expansion and non-expansion groups. Texture-based models significantly outperformed traditional predictors. GLCM achieved an AUC of 0.90 (accuracy 85.2%), GLRLM had an AUC of 0.86, and GLDM had an AUC of 0.83, all exceeding clinical and conventional imaging models (AUC ≈ 0.66–0.72). These findings highlight early texture heterogeneity as a sensitive biomarker of post-EVAR aneurysm expansion [19].

### 4.3. Prediction of Clinical Outcomes After Evar

Texture-based radiomics enables an improved prediction of clinical outcomes after EVAR by capturing aneurysm heterogeneity beyond conventional imaging metrics. Studies demonstrate that radiomic features from CTA can accurately stratify risk, predict sac behavior and severe adverse events, and identify distinct post-EVAR phenotypes using supervised and unsupervised machine learning approaches.

Huang et al. developed a texture-focused radiomics machine learning model using preoperative CTA to predict EVAR prognosis in 164 AAA patients. Patients were classified into sac shrinkage (good prognosis) and stable (poor prognosis) groups after EVAR, with no significant demographic differences. Radiomic texture features were extracted from both AAA wall and perivascular adipose tissue (PVAT), and data were split into 80% training and 20% testing sets. A support vector machine identified five AAA texture features (reflecting gray-level nonuniformity, run-length patterns, and entropy), achieving AUCs of 0.86 (training) and 0.77 (test). A 7-feature PVAT texture model reached an AUC of 0.76–0.78. Combining AAA and PVAT texture heterogeneity yielded the best performance (AUC 0.93 training; 0.87 test), highlighting texture synergy for EVAR outcome prediction [15].

Wang et al. compared texture-driven radiomic models with morphological and deep learning (DL) approaches for predicting outcomes after EVAR. In 979 patients (2010–2019; mean age 69.9 years, mean follow-up 54 months), 307 cases (31.4%) developed severe adverse events. For multimodal analysis, 493 patients were split into 80% training and 20% testing sets. Radiomics captured high-order texture patterns of AAA and intraluminal thrombus, reflecting heterogeneity and intensity variation beyond shape metrics. The radiomic logistic regression model achieved the best performance (AUC 0.93, accuracy 0.86, F1 score 0.91), outperforming the optimized morphological model (AUC 0.62) and the DL model (AUC 0.82). DCNN segmentation showed a high spatial accuracy (IoU > 90.78%), supporting reliable texture extraction [21].

Wang et al. developed a texture-focused radiomics model to predict EVAR outcomes in abdominal aortic aneurysm patients. In a retrospective cohort of 493 patients (mean follow-up 32 months), 156 cases (31.6%) experienced EVAR-related severe adverse events. From 1223 radiomic features per CTA, largely reflecting texture heterogeneity, gray-level distribution, and spatial complexity, 30 robust features were selected using Pearson correlation, ANOVA, and LASSO. Data were split into 80% training and 20% testing sets. Among multiple ML algorithms, logistic regression performed best, achieving an AUC of 0.93, accuracy of 0.86, and F1 score of 0.91. Calibration was good (*p* > 0.05), and a decision curve analysis (threshold 0.32) confirmed the clinical utility of texture-derived radiomics for EVAR risk stratification [22].

Wang et al. applied texture-based radiomics with unsupervised machine learning to identify clinical phenotypes after EVAR. From 1785 AAA patients, 1180 met inclusion criteria, with 353 patients (29.9%) developing EVAR-related severe adverse events during follow-up. Using Pyradiomics, 1223 radiomic features capturing texture heterogeneity, gray-level distribution, and spatial complexity were extracted per patient. Feature screening retained 23 texture-dominant radiomic features, which were used for clustering. In the training cohort (*n* = 944), three distinct clusters were identified, sharing similar clinical and morphological characteristics but showing marked differences in texture patterns. Kaplan–Meier analysis demonstrated significantly different freedom-from-SAE rates among clusters (training *p* = 0.0216; test *p* = 0.0253), confirming texture-derived radiomics as discriminative markers of EVAR outcomes [23].

## 5. Limitations

In addition, this work was designed as a narrative review supported by a structured literature search rather than a comprehensive systematic review. Consequently, although relevant studies were included, the literature base may not be exhaustive, and the selected articles reflect a focused overview of the available evidence on CT-based radiomics and AI for post-EVAR imaging. This selective approach should be considered when interpreting the overall conclusions.

The current evidence in radiomics for post-EVAR imaging is limited by a substantial methodological heterogeneity across acquisition protocols, image reconstruction parameters, segmentation strategies, feature extraction pipelines, and AI modeling approaches, which undermines reproducibility and prevents reliable quantitative synthesis. Most available studies are retrospective and single-center and lack robust external validation, limiting generalizability [24].

Another important source of variability relates to differences in image acquisition, segmentation methods, feature selection strategies, and model development pipelines across studies. Many radiomics investigations apply heterogeneous preprocessing steps, feature reduction techniques (e.g., correlation filtering, LASSO, or recursive feature elimination), and machine learning algorithms, making the direct comparison of results challenging. In addition, variations in CT/CTA protocols and region-of-interest delineation may substantially affect extracted feature values, thereby limiting reproducibility across institutions. Moreover, the absence of standardized validation frameworks, including nested cross-validation and independent external testing cohorts, increases the risk of model overfitting and overly optimistic performance estimates. Collectively, these methodological inconsistencies hinder clinical translation and underscore the need for greater standardization, as well as adherence to reporting frameworks such as the Radiomics Quality Score (RQS) and the TRIPOD guidelines for predictive modeling.

Technical variability remains insufficiently addressed. In particular, the inconsistent reporting of IBSI compliance, including standardized feature definitions, intensity discretization methods, voxel resampling, and preprocessing workflows, limits methodological transparency and cross-study comparability. Similarly, the application of harmonization techniques (e.g., ComBat) in multicenter datasets is rarely specified, raising concerns that scanner- or center-related batch effects may influence radiomic signatures and inflate model performance. Furthermore, the limited discussion of inter-scanner variability, including differences in CT vendors, reconstruction kernels, slice thickness, acquisition parameters, and contrast phase, reduces confidence in feature robustness. Finally, segmentation variability remains insufficiently explored, with scarce reporting of intra- and inter-observer reproducibility analyses or comparisons between manual, semi-automatic, and automated approaches, despite segmentation being a major determinant of radiomic stability. Radiomics studies are also vulnerable to overfitting and data leakage when validation strategies are not properly nested. To enhance reliability and clinical translation, standardized workflows, harmonization strategies, rigorous external validation, calibration assessment, and transparent reporting are essential.

Another limitation is the limited robustness and standardization of radiomic features across studies, as differences in preprocessing, feature extraction pipelines, and inconsistent adherence to standardization initiatives (e.g., IBSI) may affect the reproducibility and comparability of results.

Another important source of variability relates to differences in feature selection strategies and model development pipelines across studies. Many radiomics investigations apply different preprocessing steps, feature reduction techniques (e.g., correlation filtering, LASSO, or recursive feature elimination), and machine learning algorithms, making the direct comparison of results challenging. Moreover, the absence of standardized validation frameworks, including nested cross-validation and independent external testing cohorts, increases the risk of model overfitting and optimistic performance estimates. These methodological inconsistencies highlight the need for adherence to emerging reporting standards such as the Radiomics Quality Score (RQS) and the TRIPOD guidelines for predictive modeling.

In addition, the heterogeneity of imaging phases and CT acquisition protocols across studies (e.g., contrast phase, scanner vendor, slice thickness, reconstruction kernel), which may influence radiomic feature extraction and reduce the comparability between studies.

Furthermore, the variability in tumor segmentation strategies across studies (manual or semi-automatic) and the limited assessment or reporting of inter- and intra-observer reproducibility, which may affect the stability of extracted radiomic features.

Likewise, the variability in model development methodologies across studies, including differences in feature selection procedures, machine learning algorithms, and validation strategies, which may influence model performance and limit reproducibility.

Finally, that many radiomics models were validated only internally, with relatively few studies performing external validation, which may limit the generalizability and clinical applicability of the proposed models.

Moreover, the risk of overfitting should be considered, as many radiomics models are developed using high-dimensional feature sets in relatively small cohorts, which may lead to overly optimistic performance estimates.

## 6. Clinical Translation and Potential Impact

Radiomics-based risk stratification may support a shift from uniform post-EVAR imaging schedules to risk-adapted surveillance. In particular, models developed on unenhanced CT may be clinically relevant in patients with reduced renal function or high cumulative contrast exposure, potentially decreasing the reliance on repeated contrast-enhanced CTA in selected low-risk individuals. Conversely, high-risk radiomic signatures (e.g., increased heterogeneity/entropy or PVAT texture alterations) could identify patients requiring intensified monitoring, earlier CTA, or closer evaluation for endoleak and sac expansion. For clinical adoption, future studies should explicitly report how model outputs translate into actionable decisions (e.g., recommended follow-up interval, trigger thresholds for CTA/reintervention) and evaluate whether radiomics-guided strategies improve patient outcomes compared with standard surveillance.

## 7. Conclusions and Future Perspectives

The available literature suggests that CT-based texture radiomics may provide quantitative markers of aneurysm heterogeneity beyond the conventional morphological measurements used in routine post-EVAR surveillance. Radiomic signatures derived from the aneurysm sac, intraluminal thrombus, and perivascular adipose tissue have shown potential for endoleak detection, the prediction of aneurysm sac behavior, and risk stratification for EVAR-related adverse events. However, these findings should be interpreted cautiously, as the current evidence base remains limited by the small number of studies, substantial methodological heterogeneity, lack of standardization across imaging acquisition and analysis workflows, limited external validation, and uncertain reproducibility across centers and scanners.

Future research should focus on multicenter prospective validation, standardized radiomics workflows, and harmonization techniques to improve reproducibility. The integration of radiomics with clinical variables, laboratory biomarkers, genomics, and hemodynamic data may further enhance predictive performance. Explainable AI models and automated segmentation pipelines will be essential for clinical adoption. Ultimately, texture-based radiomics has the potential to support precision imaging and decision support systems, transforming post-EVAR surveillance from reactive monitoring to proactive risk stratification.

Compared with previous broader reviews addressing artificial intelligence across multiple vascular diseases and imaging modalities, the present work provides a focused synthesis specifically dedicated to CT-based texture radiomics in the post-EVAR setting. By concentrating on this defined clinical context, this review highlights how radiomic biomarkers derived from CT and CTA can contribute to clinically relevant tasks such as endoleak detection, the prediction of aneurysm sac behavior, and the stratification of EVAR-related adverse outcomes. This targeted perspective helps clarify the current evidence base and underscores the emerging role of texture radiomics as a potential decision-support tool for risk-adapted post-EVAR surveillance.

In addition, several studies included relatively small patient cohorts, which may affect the robustness and generalizability of the reported radiomics models.

The main insight emerging from the available literature is that CT-based texture radiomics may provide quantitative markers of aneurysm heterogeneity beyond the conventional morphological measurements used in routine post-EVAR surveillance. Across the studies reviewed, this potential appears most relevant in three areas: endoleak detection, the prediction of aneurysm sac behavior, and risk stratification for EVAR-related adverse events. Taken together, these findings suggest that texture-based radiomics may offer added value over conventional imaging assessment by capturing subtle tissue-related information not reflected by sac diameter alone, although the current evidence remains preliminary and heterogeneous.

Key Clinical Points

- Texture-based CT radiomics enables the quantitative assessment of aneurysm sac, intraluminal thrombus, and perivascular adipose tissue heterogeneity beyond conventional morphological measurements after EVAR.
- Radiomics and AI models demonstrate good to excellent performance for predicting endoleaks, aneurysm sac behavior, and EVAR-related severe adverse events, outperforming morphology-only assessment.
- Radiomics derived from non-contrast CT may reduce the reliance on contrast-enhanced CTA, supporting safer surveillance in selected high-risk patients.
- The integration of radiomics with clinical and imaging variables may facilitate personalized, risk-adapted post-EVAR follow-up and the earlier identification of patients requiring intensified monitoring or reintervention.

## Figures and Tables

**Table 1:** Summary of studies investigating texture-based radiomics and artificial intelligence for outcome prediction and surveillance after EVAR.

Author (Year)	Design	N	Modality	Radiomic Target	AI/ML Method	Outcome	Main Performance
**Huang 2025 [15]**	Retrospective	164	CTA	AAA wall, PVAT	SVM	Sac shrinkage vs. stability	Combined AUC 0.87 (test)
**Lv 2024 [16]**	Retrospective	79	CTA	PVAT	Logistic regression	Aneurysm growth	AUC 0.78
**Yang 2024 [17]**	Retrospective	167	Non-contrast CT + CTA	Aneurysm sac	DL	Endoleak detection	AUC 0.951
**Hu 2023 [18]**	Retrospective	216	Non-contrast CT	Aneurysm sac	Multiple ML	Endoleak detection	Mean AUC 0.86
**Charalambous 2022 [10]**	Prospective	(Reported in study)	CTA	T2EL region/sac	SVM	Sac expansion	AUC 0.955 (6 mo)
**Ding 2020 [19]**	Retrospective	99	CT	Aneurysm sac	MLP	Expansion	AUC 0.90
**García 2012 [20]**	Retrospective	(Reported in study)	CTA	ILT	Texture classifiers	Endoleak-related evolution	Accuracy 93.4%
**Wang 2022 [21]**	Retrospective	979	CTA	AAA, ILT	LR/SVM/DL	SAEs	AUC 0.93
**Wang 2023 [22]**	Retrospective	493	CTA	AAA ± ILT	Logistic regression	SAEs	AUC 0.93
**Wang 2025 [23]**	Retrospective	1180	CTA	Sac, ILT	Unsupervised ML	SAEs	Significant cluster differences

Abbreviations: AAA, abdominal aortic aneurysm; AI, artificial intelligence; AUC, area under the curve; CTA, computed tomography angiography; CT, computed tomography; DL, deep learning; EVAR, endovascular aneurysm repair; ILT, intraluminal thrombus; LR, logistic regression; ML, machine learning; MLP, multilayer perceptron; PVAT, perivascular adipose tissue; SAE, severe adverse event; SVM, support vector machine.

## Data Availability

Not applicable. No new data were created or analyzed in this study.

## Abbreviations

AAA	Abdominal aortic aneurysm
AI	Artificial intelligence
ANOVA	Analysis of variance
AUC	Area under the curve
CI	Confidence interval
CT	Computed tomography
CTA	Computed tomography angiography
DCNN	Deep convolutional neural network
DL	Deep learning
EVAR	Endovascular aneurysm repair
GLCM	Gray-level co-occurrence matrix
GLDM	Gray-level dependence/difference matrix
GLRLM	Gray-level run length matrix
HU	Hounsfield unit
ILT	Intraluminal thrombus
IoU	Intersection over Union
LASSO	Least absolute shrinkage and selection operator
ML	Machine learning
MLP	Multilayer perceptron
PVAT	Perivascular adipose tissue
RF	Radiomic features
ROI	Region of interest
SAE	Severe adverse event
SVM	Support vector machine
T2EL	Type 2 endoleak
